# IVIG response in pediatric acute-onset neuropsychiatric syndrome correlates with reduction in pro-inflammatory monocytes and neuropsychiatric measures

**DOI:** 10.3389/fimmu.2024.1383973

**Published:** 2024-10-03

**Authors:** Isaac Melamed, Shamma Rahman, Heather Pein, Melinda Heffron, Jennifer Frankovich, Huub Kreuwel, Elizabeth D. Mellins

**Affiliations:** ^1^ IMMUNOe Research Centers, Centennial, CO, United States; ^2^ Department of Pediatrics, Stanford University School of Medicine, Stanford, CA, United States; ^3^ Scientific and Medical Affairs, Octapharma USA, Paramus, NJ, United States

**Keywords:** pediatric acute-onset neuropsychiatric syndrome, PANS, pediatric autoimmune neuropsychiatric disorder associated with streptococcal infections, PANDAS, intravenous immunoglobulin, IVIG, autoimmune

## Abstract

**Introduction:**

Pediatric Acute-Onset Neuropsychiatric Syndrome (PANS) is characterized by abrupt onset of obsessive-compulsive disorder or eating restriction along with the abrupt onset of other co-occurring symptoms (tics, behavioral and cognitive regression, *etc.*). PANS is thought to be a post-infectious immunopsychiatric disorder, although as with most post-infectious disorders, it is challenging to establish a causal relationship with proposed infectious triggers. Intravenous immunoglobulin (IVIG) can modulate inflammation and support the elimination of infection and has been used for treatment of many post-infectious inflammatory disorders and autoimmune conditions. The aim of the study is to explore the pro-inflammatory state in PANS before and after administration of IVIG.

**Methods:**

Children with moderate-to-severe PANS received six infusions of IVIG (Octagam 5%, Octapharma) every 3 weeks with post treatment follow-up. Blood samples and psychiatric measures were obtained at Visits 1 (pre-treatment), 7 and 8 (4 and 11 weeks after last infusion, respectively). Myeloid cell activation was assessed via flow cytometry.

**Results:**

All ten patients included in the study were male, White, with mean age 12.4 years (range 6–16). Statistically significant improvements following IVIG treatment were demonstrated in all psychometric assessments and parent questionnaires including CY-BOCS (obsessive compulsive scale), YGTSS (tic scale) and a parent PANS rating scale (for all scales p<0.001). The fraction of pro-inflammatory monocytes and dendritic cells decreased from pre-IVIG treatment levels. The proportional reductions were not compensated by increases in total white blood cells; pro-inflammatory monocytes post-IVIG were decreased as a proportion of CD14+ myeloid cells and in absolute number.

**Conclusions:**

The results of this study suggest that active PANS is associated with a pro-inflammatory state. This pro-inflammatory profile and psychometric scores improved following IVIG treatment. Future work will aim to further elucidate the roles of innate and adaptive immune responses in PANS and the regulatory mechanism(s) of IVIG in PANS treatment.

## 1 Introduction

Pediatric Acute-Onset Neuropsychiatric Syndrome (PANS) is a diagnostic criterion created to describe children with sudden onset of obsessive-compulsive symptoms and/or eating restriction along with other severe neuropsychiatric symptoms (including tics). Due to the suddenness of onset, many clinicians and researchers hypothesize an infectious or trauma-related trigger (1–3).

Pediatric Autoimmune Neuropsychiatric Disorder associated with Streptococcal infections (PANDAS) was initially defined in the late 1990s to describe the subset of children with rapid escalation of obsessive-compulsive symptoms and/or tics following group A streptococcal infections (GAS) (4). Since many patients come to clinical attention after the window of opportunity to diagnose GAS, and since other infections (*Mycoplasma pneumonia*, Epstein-Barr virus, influenza, etc.) may theoretically trigger a similar pro-inflammatory state impacting the brain, PANS diagnostic criteria, agnostic to hypothesized triggers/infections, were proposed (1). Recently, cases of SARS-CoV-2 related PANS have been reported as well (5). Additionally, a recent study showed that life events such as environmental, family, and social changes can exacerbate the clinical condition or generate new symptoms in young patients (6).

PANS shares features with Sydenham’s chorea, a neurologic manifestation of an inflammatory response to a GAS (4). These disorders have a considerable overlap in their clinical presentation and pathophysiology including prominent obsessive-compulsive symptoms, irritability, emotional dysregulation, irrational fears, outbursts of inappropriate behavior, night terrors, and personality changes as well as changes in basal ganglia as shown in imaging studies (7–13). The variety of neuropsychiatric symptoms in Sydenham’s chorea and PANDAS/PANS was hypothesized to be caused by an underlying dysfunction of basal ganglia as a result of an immune response to the infection and subsequent inflammation and/or misdirected autoantibodies altering neuronal functions (14). Additionally, similar antineuronal antibodies and responses to immunomodulatory treatments were demonstrated in both disorders (15–19).

In current clinical practice, a PANS diagnosis is based on history and physical examination with a focus on symptoms rather than the cause, as causal relationships cannot be established in individual patients. Current treatment of PANS is three pronged: 1) treatment of infection if still present at the time of presentation and clearance of GAS from the household; 2) anti-inflammatory treatments; and 3) psychiatric and behavioral interventions. A systematic review of PANS treatment suggested that the role medical therapy in PANS remains controversial due to lack of established protocol and variable response depending on the drug used and timing of administration (20). The use of nonsteroidal anti-inflammatory drugs (NSAIDs) is the first line of anti-inflammatory treatment (16). In the setting of ongoing moderate-severe symptoms current treatment recommendations suggest the use of corticosteroids for moderate to severe PANS. However, IVIG is recognized as the preferred treatment for these patients by most members of the PANS Research Consortium, particularly in the case of a potential ongoing occult infection (16).

IVIG is a concentrated pooled preparation of normal human immunoglobulins obtained from human plasma of several thousand healthy donors manufactured by the cold ethanol fractionation process followed by ultrafiltration and chromatography. Pooling provides a diversity of antibody repertoires and specificities. IVIG can modulate inflammation and support the elimination of infection and has been used for treatment of post-infectious inflammatory disorders and autoimmunity (21, 22). In previous studies, IVIG has been shown to significantly decrease symptom severity in children with infection-triggered OCD and tic disorders (23) and moderate to severe PANDAS (24). We have previously performed an open-label study of 21 patients with moderate or severe PANS treated with six consecutive IVIG infusions (25). The results indicated that IVIG treatment was followed by a statistically significant improvement in psychometric scores, with sustained benefits for at least 8 weeks, and up to 46 weeks in a subset of participants (25).

However, additional data are necessary to strengthen the conclusion that PANS is an immune-mediated inflammatory disease as well as to understand how the IVIG treatment exerts its immunomodulatory effect. This study aimed to expand on this understanding by exploring immune dysregulation in PANS and its potential amelioration with IVIG therapy.

## 2 Methods

### 2.1 Participants and study design

This was an open-label study conducted at three clinical/research sites in the United States: IMMUNOe Research Centers (Centennial, CO); Midlands Pediatrics (Papillion, NE); and Allergy, Asthma & Immunology Relief Research Institute (Charlotte, NC). The study was approved by a central Institutional Review Board (Advarra Columbia, MA). The parents of participants provided informed consent, and assent was obtained from all study participants.

**Figure 1:**
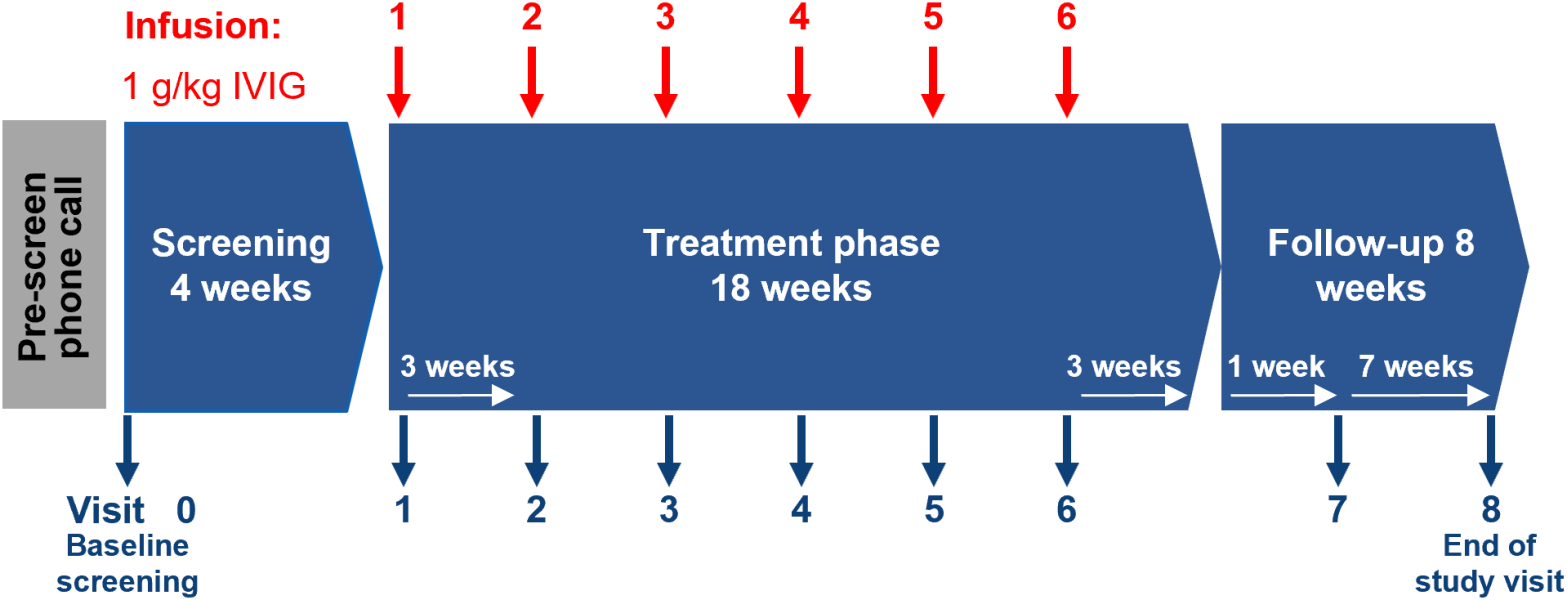
Study design. IVIG, intravenous immunoglobulin; PANS, pediatric acute-onset neuropsychiatric syndrome.

Study design is shown in **Figure 1**. Children aged 4–16 years old with moderate-to-severe PANS were eligible for the study. PANS was diagnosed according to the following criteria: 1) Abrupt, dramatic onset of obsessive-compulsive disorder or severely restricted food intake; 2) Concurrent presence of at least two additional neuropsychiatric symptoms, with similarly severe and acute onset, specifically: anxiety, emotional lability/depression, irritability/aggression/oppositional behaviors, behavioral regression, deterioration in school performance, sensory/motor abnormalities, somatic signs and symptoms and; 3) Symptoms that were not better explained by a known neurologic or medical disorder, such as Sydenham chorea, systemic lupus erythematosus, Tourette disorder or others (1). Exclusion criteria included previous IVIG therapy within the last 4 months, allergic reactions to blood products, other psychiatric co-morbidities outside of expected PANS diagnoses (including severe autism) and patients who, in the investigator’s opinion, might not be suitable for the trial. Study participants had been receiving their standard of care therapy for up to one year before enrollment and during the screening period. None of the participants were on ongoing psychotherapy treatment.

The study had a pre-screen phone call followed by a 4-week screening/baseline phase. The treatment phase lasted for 18 weeks and patients received 1 g/kg body weight of IVIG (Octagam 5%, Octapharma AG) at Visits 1–6 (i.e., every 21 ± 3 days for a total of 6 infusions). Patients were followed up at Visit 7 (4 weeks after last infusion) and 8 (end-of-study visit [EOS], 11 weeks after last infusion/7 weeks after Visit 7). Psychiatric assessments were performed at Visits 1, 7 and 8 using validated psychometric scales, the Children’s Yale-Brown Obsessive-Compulsive Scale (CY-BOCS) and Yale Global Tic Severity Scale (YGTSS). In addition to these assessments, two parent-rated questionnaires were utilized during the study; the PANS Symptom Scale was used as a pre-screening measure to validate the PANS diagnosis and following the IVIG treatment to assess treatment efficacy (26). Additionally, the Parent-Rated PANS Questionnaire (PRPQ) was completed at all Visits 1–8. Blood samples for laboratory analysis were taken at Visits 1 (pre-treatment), 7 and 8.

### 2.2 Outcomes

The primary study objectives were to assess the effect of IVIG therapy on monocyte activation in the laboratory, and psychological and behavioral clinical evaluations in patients with PANS.

### 2.3 Psychological evaluations/behavioral assessments

The PANS Symptom Scale (26) was used to validate the PANS diagnosis and to provide a baseline measurement of disease severity. Following the IVIG treatment, this scale was used to assess the treatment efficacy. The scale was developed by Swedo et al. at the National Institute of Mental Health and it includes the symptoms from the PANS criteria scored from 0–5 (none to very severe). The total score ranges from 0 to 100 and consists of the OCD part (0–25), the associated neuropsychiatric symptom part (0–25), and the impairment part (0–50) (26). The ratings were based on parent interviews.

CY-BOCS is a widely used and validated tool designed to rate the severity and type of symptoms in patients with OCD (27). The scale rates the characteristics of obsessive or compulsive ideation and actions on a scale of 0 (none) to 4 (extreme) and yields a total severity score between 0 (no symptoms) and 40 (severe). YGTSS is the most widely used scale to quantify tics (28). The rating is based on a clinician-rated, semi-structured interview that begins with a systematic inquiry of tic symptoms in the preceding week. Current motor and phonic tics are then rated separately according to number, frequency, intensity, complexity, and interference. The global severity score ranges from 0 (no symptoms) to 100 (most severe).

The Parent-Rated PANS Questionnaire (PRPQ) is 58-item tool that was developed for our previous study (25). The questionnaire was used to evaluate parent/caregiver’s assessment of symptom severity and was filled out by the parent/caregiver at each study visit. The answers to the questions are graded based on severity (0 - none, 1 - mild, 2 - moderate, 3 - severe, 4 - extreme).

### 2.4 Monocyte activation assessment

Change in activation state of circulating myeloid cells in children with PANS from baseline (pre-treatment, Visit 1) to post-treatment (Visits 7, 8) was evaluated by flow cytometry (FACS) according to a previously established protocol (29). Briefly, freshly collected blood samples were immediately fixed ex vivo using SmartTube fixative and stored at -80°C until ready for use. Once all samples were gathered, frozen fixed whole blood samples were sequentially thawed, first at 4°C cold room for 20 minutes and next in a 15°C water bath for another 20 minutes. Thawed samples were incubated with Thaw-Lyse buffer (SmartTube Inc.) for 10 minutes and subjected to centrifugation at 1200 rpm for 10 minutes at room temperature. Supernatant was discarded and pellet was dissolved in additional Thaw-Lyse buffer to remove all red blood cells. The lysing step was repeated three times in total and the final pellet was resuspended in FACS buffer. Samples were prepared for flow cytometric analysis, as described in the published protocol (29). All antibodies were purchased from BioLegend (San Diego, CA, USA) and were used at the concentration specified by the manufacturer. Details of the antibody panel are included in **Supplementary Table 1** and the gating strategy for inflammatory monocytes and pro-inflammatory dendritic cells are shown in **Supplementary Figure 1**.

Immune cells were identified using manual gating of FACS dotplots, based on the expression of cell lineage-specific markers. T cells, B cells, granulocytes, and red blood cells were excluded, based on the expression of CD3, CD19, CD66b, and CD235a, respectively. Myeloid cells were gated based on their expression of HLA-DR and three monocyte subsets were identified using plots of CD14 versus CD16 (two major markers of monocytes), whereas the non-monocytic population (CD14-CD16-) contained most of the dendritic cells. Proinflammatory CD14+ dendritic cells were identified within the HLA-DR+CD14+ cell population, as CD14 is a shared marker between monocytes and this subset of dendritic cells (30). To identify proinflammatory polarized monocytes, CD14+ monocytes were further gated based on increased levels of CD64 and CD86, commonly used markers for proinflammatory (M1) macrophages (31). Cells that were HLA-DR+CD14+CD64+CD86+ were classified as proinflammatory monocytes. Flow cytometry data were analyzed by FlowJo (FlowJo, LLC, Ashland, OR, USA) v.10 software.

### 2.5 Statistical analysis

Unadjusted descriptive statistics were conducted to summarize the endpoints for eligible participants. Mean, standard deviation (SD) was used to describe outcomes of continuous variables, and percentages for categorical variables. Changes from baseline to follow-up visits were tested using Student’s t test for continuous variables and Fisher’s exact tests for categorical variables. A two-sided p-value <0.05 was considered statistically significant. All analyses were conducted using SAS 9.4 software (SAS Institute, Cary, NC). Statistical analysis of the flow cytometry data was performed using GraphPad Prism v8 software (GraphPad Software, Inc., San Diego, CA). Paired samples were analyzed by non-parametric Mann-Whitney test with a p value <0.05 indicating significance.

## 3 Results

### 3.1 Study participants

A total of 16 patients were screened, and 10 patients were enrolled and completed the study. Six patients did not meet the study inclusion criteria. All 10 enrolled patients were male, White, with mean age of 12.4 years (range 6–16). Baseline characteristics of the patients are summarized in **Table 1**. Eight patients had been diagnosed with PANS more than 2 years prior to screening. PANS Symptom Scale score at baseline was high (mean 39.7, SD 6.25). Inflammatory marker C-reactive protein levels, as well as standard laboratory safety markers, were all in the normal range.

**Table 1:** Demographics and baseline clinical characteristics.

Characteristic	Mean (SD)
Age at start of the study	12.4 (3.92)
Sex, male, n (%)	10 (100)
Race, White n (%)	10 (100)
Weight at Visit 1, kg	49.6 (14.38)
PANS Symptoms Scale score (0–100)	39.7 (6.25)
CY-BOCS (0–40)	25.5 (3.62)
YGTSS (0–100)	55.5 (20.67)
Parent-rated PANS scale	78.2 (24.3)

In all behavioral scales, higher scores represent more severe symptoms.

CY-BOCS, Children’s Yale-Brown Obsessive-Compulsive Scale; PANS, pediatric acute-onset neuropsychiatric syndrome; SD, standard deviation; YGTSS, Yale Global Tic Severity Score.

### 3.2 Behavioral assessments

**Figure 2:**
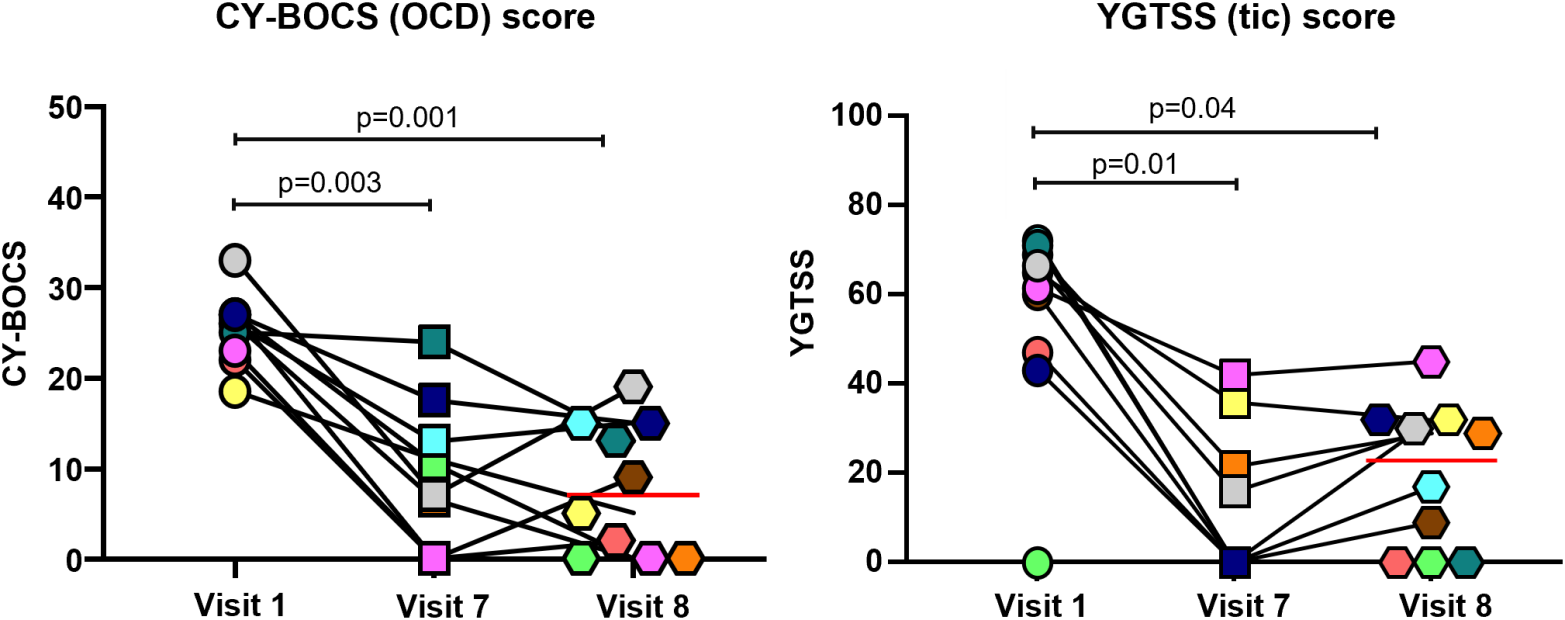
Changes in CY-BOCS and YGTSS scores in patients with PANS following IVIG infusion. Each color represents a single patient. Red line represents mean value. P values calculated from analysis of paired samples. CY-BOCS, Children’s Yale-Brown Obsessive-Compulsive Scale; IVIG, intravenous immunoglobulin; PANS, pediatric acute-onset neuropsychiatric syndrome; YGTSS, Yale Global Tic Severity Score.

**Figure 3:**
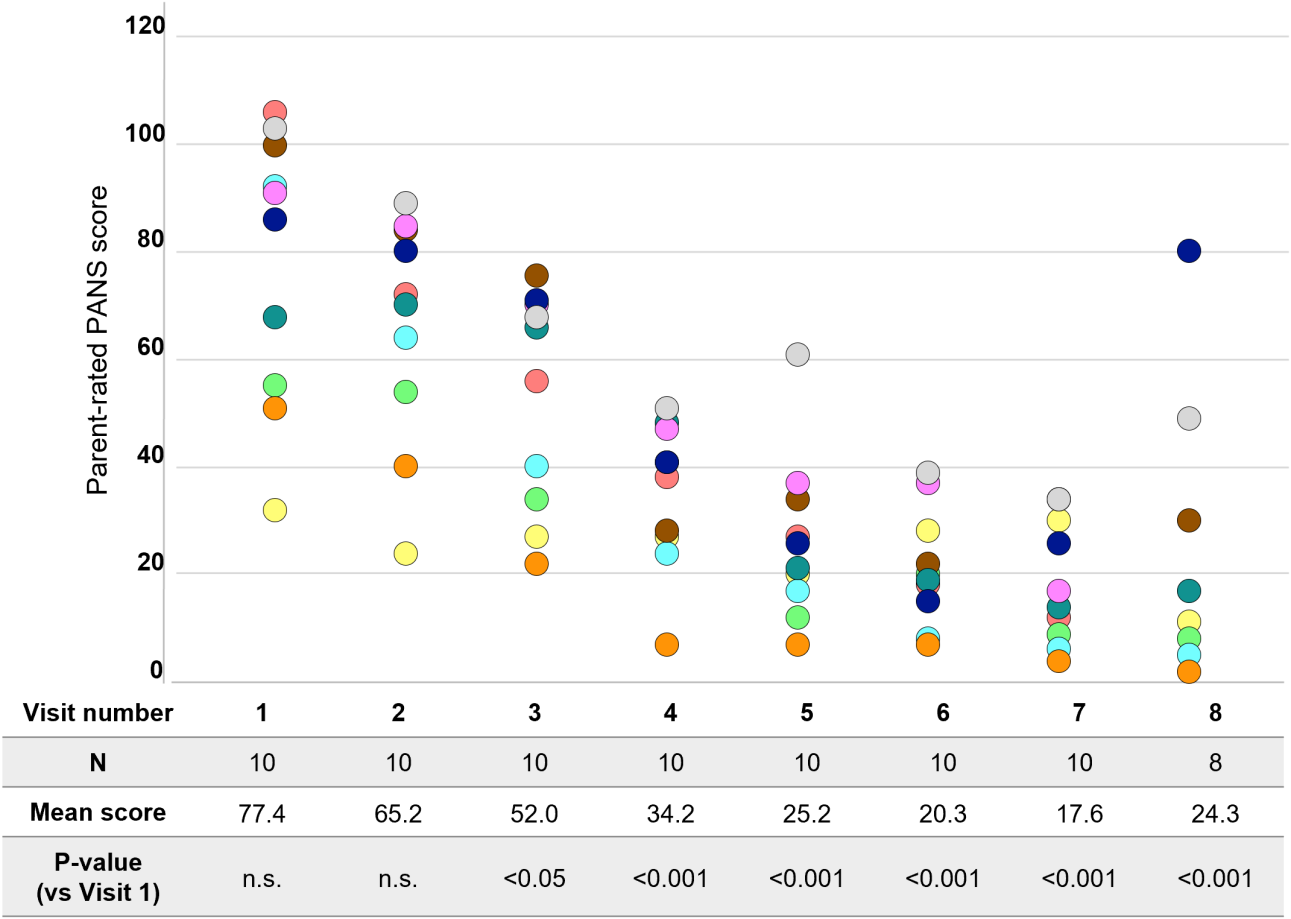
Change in parent-rated PANS questionnaire scores. Each color represents a single patient. PANS, pediatric acute-onset neuropsychiatric syndrome; n.s., not significant.

Statistically significant improvements from baseline to end of treatment and in the follow-up visits were demonstrated in all psychometric assessments and parent questionnaires (**Figures 2**, **3** and **Table 2**). PANS Symptoms Scale score decreased by 65.1% in Week 7 (p<0.001) and 44.8% in Week 8 (p=0.006). CY-BOCS scores in these patients improved significantly from Baseline to Visits 7 and 8 after IVIG infusion (p=0.001 and p=0.003, respectively). Statistically significant improvements after IVIG infusion were similarly demonstrated in the YGTSS scores (p=0.001 at Visit 7 and p=0.0001 at Visit 8). Interestingly, in some patients, the scores started to rise again by Visit 8 (i.e., 11 weeks after the last infusion).

**Table 2:** Changes in behavioral assessments.

Assessment	Visit 7	Visit 8
**PANS Symptoms Scale (n [%])**	7 (70)	10 (100)
Percent change from baseline	-65.1%	-44.8%
Mean change	-25.84	-17.81
*p* values	**<0.001**	**0.006**
**CY-BOCS (n [%])**	10 (100)	10 (100)
Percent change from baseline	-64.83	-69.1
Mean change	-16.29	-17.44
*p* values	**<0.001**	**<0.001**
**YGTSS (n [%])**	10 (100)	10 (100)
Percent change from baseline	-79.19	-65.04
Mean change	-43.45	-36.1
*p* values	**<0.001**	**<0.001**
**Parent-Rated PANS Questionnaire (n [%])**	10 (100)	8 (80)
Percent change from baseline	-77	-69.31
Mean change	-68.2	-54.2
*p* values	**<0.001**	**<0.001**

Bold text indicates statistically significant change from baseline (p<0.05). CY-BOCS, Children’s Yale-Brown Obsessive-Compulsive Scale; PANS, pediatric acute-onset neuropsychiatric syndrome; YGTSS, Yale Global Tic Severity Score.

PRPQ is the only measure that was evaluated at all visits and thus provides interim treatment efficacy evaluation. Parents reported significant (p<0.05) reduction in PANS symptoms from Visit 3 onwards (i.e., after 2 infusions) (**Figure 3**). The score decreased by 68.2% in Week 7 and 54.2% in Week 8 (both p<0.001).

### 3.3 Proinflammatory cell levels

**Figure 4:**
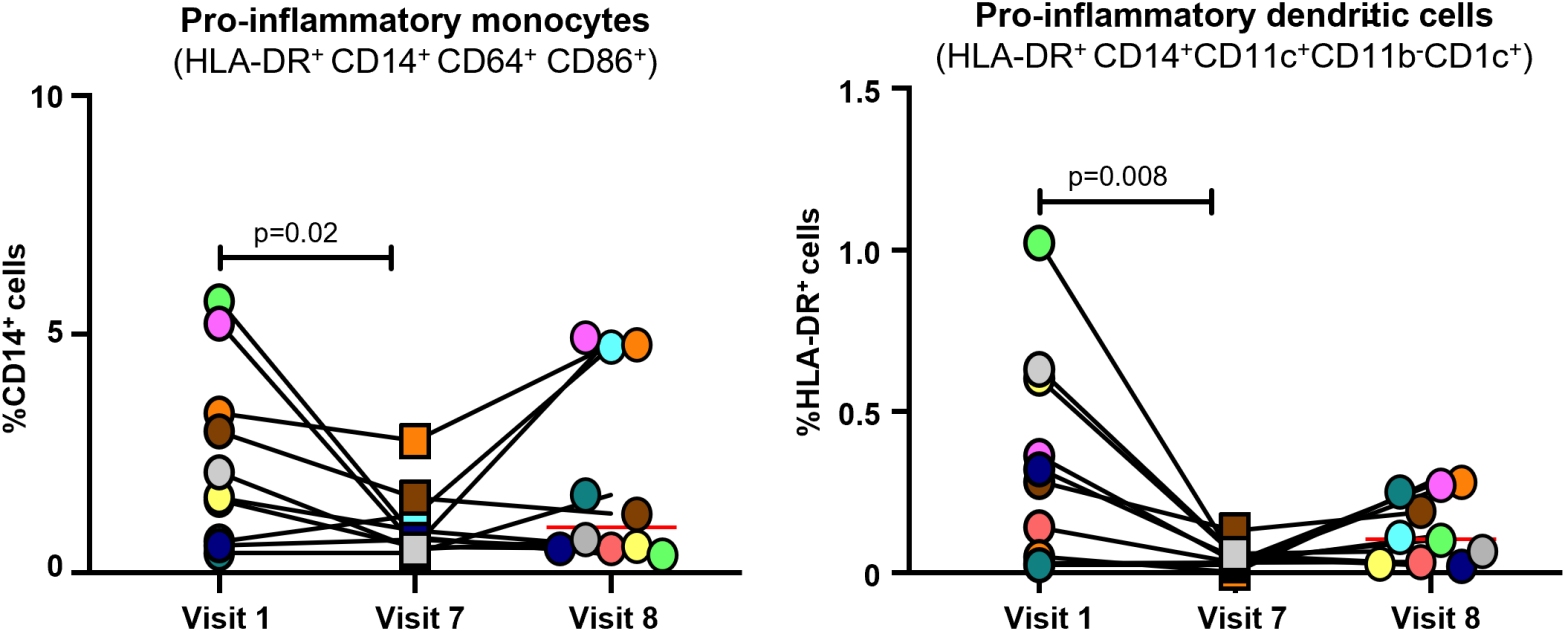
Levels of pro-inflammatory cells in patients with PANS following IVIG infusion. Each color represents a single patient. Red line represents mean value. Paired samples were analyzed by non-parametric Mann-Whitney test with a P value <0.05 indicating significance. IVIG, intravenous immunoglobulin; PANS, pediatric acute-onset neuropsychiatric syndrome.

Proportions of activated inflammatory myeloid cells (monocytes and dendritic) cells after the IVIG treatment significantly decreased compared to pre-treatment values in all patients by Visit 7 (**Figure 4**). In three patients, a slight increase in the proportion of pro-inflammatory monocytes was observed by Visit 8 (i.e., 11 weeks after the last infusion). Notably, these patients also showed an increase in total WBC count, whereas those patients whose pro-inflammatory cell proportions remained low did not show an increase in total WBC count, indicating the absolute numbers of these cells were lower than pre-IVIG (**Supplementary Table 2**).

## 4 Discussion

This open label study showed that six infusions of IVIG was followed by improvement in psychometric scores in PANS patients, including OCD and tics. Additionally, for the first time, data showing a decrease in pro-inflammatory monocytes and dendritic cells in these patients after the completion of IVIG treatment were presented.

The results of this study are in line with prior reports of the effects of IVIG on reducing symptoms in PANS/PANDAS patients in both randomized control trials and observational studies. In 1999, the results of the first placebo-controlled study using IVIG treatment to treat post-GAS neuropsychiatric symptoms (OCD and tics) were published (23). The study compared IVIG and plasma exchange versus placebo and demonstrated that both treatments were associated with symptom improvement (23). In the next placebo-controlled trial, published in 2016, 35 patients meeting the PANDAS criteria received two consecutive doses of IVIG or placebo, followed by optional open-label treatment for nonresponding patients (24). The mean decrease in OCD severity was greater in the IVIG group than in the placebo group; however, between-group differences were smaller than anticipated, and the double-blind comparison failed to demonstrate superiority of IVIG over placebo (24). The nonresponding patients who received open-label IVIG after the initial 6 week blinded phase showed improvement in the OCD scores from baseline by 55% and 62% at Week 12 and 24, respectively (24).

In our previous open-label study, we demonstrated that six consecutive infusions of 1 g/kg IVIG every three weeks can be an efficacious treatment for PANS patients. Importantly, we observed sustained benefits for at least 8 weeks after completion of the infusions, and up to 46 weeks in a subset of participants (25). Significant improvements in the mean CY-BOCS, YGTSS and the parent-rated PANS symptom scale versus baseline were observed at early follow-up visits (25). In a subset of participants in a late follow-up visit (29–46 weeks following the final infusion), results indicated that tics returned in some patients, although they were still below baseline levels (25). Similarly, in the current study, some of the evaluated scores, including YGTSS (tic scale score), started to rise in some patients at the last follow-up visit (11 weeks after the last infusion).

Another open-label study from Sweden by Hajjari et al. (2022) utilized three monthly 2 g/kg IVIG treatments in children with PANS and showed considerable improvements in PANS global symptoms, the PANS Symptoms Scale (ratings based on parent and patient interviews), Clinical Global Impression - Severity and Improvement (CGI-S and CGI-I) scales and the CY-BOCS scale scores improved, lasting at least one month after the treatment (32). A recently published retrospective study described children diagnosed with PANS treated with IVIG doses and showed improvement in 11/12 patients, in one or multiple investigated domains including memory (58% patients), sensory-motor (37% patients), and visual-motor integration (30% patients) (33). The efficacy of the treatment was independent of time elapsed from the disease onset, emphasizing the positive effect of immunomodulatory therapy (33).

The results of the present study thus further strengthen the correlation between IVIG treatment and patient improvement. Despite these results, high quality evidence from larger, placebo-controlled studies supporting the use of IVIG treatment in PANS is still lacking. This gap in evidence will be addressed by a superiority randomized, double-blind, placebo-controlled phase 3 trial (NCT04508530) designed to determine efficacy of IVIG treatment in PANS patients, which is currently ongoing and recruiting patients with a planned enrollment of >90 patients (34).

In the present study the effect of IVIG treatment on pro-inflammatory myeloid cell profiles in PANS patients was evaluated for the first time. The data show a decrease in pro-inflammatory monocytes and dendritic cells following six IVIG treatments. The improvements seen in the pro-inflammatory myeloid cells and the psychometric scores began to attenuate in a small subset of patients during the post-treatment follow-up period (between Visits 7 and 8), suggesting that further treatment with IVIG may be necessary to maintain a durable improved state.

Overall, the results of this study strengthen the conclusion that the active PANS state (high levels of OCD, tics, *etc.*) is associated with an inflammatory process and that the clinical symptoms and pro-inflammatory state improve with immunomodulation. The current hypothesis is that PANS is a post-infectious inflammatory disorder which may involve autoantibodies (17), microglial cell activation (11), and inappropriate release of or response to inflammatory cytokines (35). These result in an inflammatory disorder of the basal ganglia, similar to other autoimmune encephalitides, such as Sydenham’s chorea (36). In fact, a recent neuroimaging study in PANS patients confirmed microstructural changes in basal ganglia, consistent with inflammatory changes similar to Sydenham’s chorea (12).

Although a complete understanding of the immune response in PANS is still unfolding, a growing body of clinical experience and data corroborates the concept of PANS as an inflammatory disease including the high rate of familial autoimmunity and co-morbid arthritis (8, 37, 38). IVIG, as broad-spectrum immunomodulatory treatment, can therefore form part of the clinical solution for these patients, particularly those with moderate to severe disease. There are many mechanisms by which IVIG exerts its immunomodulatory and anti-inflammatory effects on both the innate and adaptive immune systems; however, its exact role in PANS remains to be determined (21). Experimental and clinical data indicate that the therapeutic benefit of IVIG therapy involves both soluble mediators as well as cellular components of the immune system (22). IVIG can affect innate immunity by interrupting the steps in the complement activation cascade and blocking Fc-receptor mediated activation of innate immune cells, such as monocytes and macrophages. Evidence from a murine model of immune thrombocytopenia suggests that the molecular basis of IVIG treatment is its ability to induce expression of the inhibitory Fcγ receptor (FcγRIIB) on macrophages and thus prevent platelet consumption (39). The protective effect of IVIG is conferred by the Fc region, but not the Fab portion of IgG (39). Studies in dermatomyositis suggest the IVIG effect might involve inhibition of complement consumption and interference with formation of the membrane attack complex, preventing capillary destruction and microangiopathy (40).

Further studies are needed to help to clarify the role of post-infectious inflammation and its impact on brain in PANS and related neuropsychiatric disorders, as well as the mechanisms of IVIG treatment in these disorders. In some PANS and OCD patients, immunodeficiency is also observed, suggesting there may be additional innate and/or adaptive immune system abnormalities contributing to post-infectious neuropsychiatric deteriorations. Understanding the mechanism of IVIG in these disorders is of particular interest as IVIG is a key approach to manage immunodeficiency syndromes. Lastly, it will be crucial in future studies to identify biomarkers that determine which patients will benefit most from IVIG treatment.

The main limitations of this open-label study are the small sample size as well as the lack of control group. To mitigate potential bias, assessments were conducted by two independent investigators and a psychologist who were not associated with the study. Although a formal control group was not included, each patient served as their own internal control, allowing us to measure changes in psychiatric symptoms and biomarkers before and after IVIG treatment. Larger randomized, blinded and placebo-controlled trials are needed to evaluate the effect of IVIG treatment on PANS. Indeed, based on the positive preliminary results documented in this and other studies, a large placebo-controlled phase 3 trial comparing the effect of IVIG versus placebo in patients with PANS is currently ongoing (NCT04508530) with estimated completion later this year. Additionally, analysis of patient blood samples may not most accurately reflect the immunological response in the central nervous system as it provides only a distant picture of the processes involved in the mechanisms of the disorder. However, affected brain tissue from active PANS patients is not available for analysis, beyond rare post-mortem samples.

## 5 Conclusions

Overall, our results support the concept of PANS as an inflammatory disease. Treatment with IVIG was associated with both a decrease in pro-inflammatory myeloid cells (monocytes and dendritic cells), and psychiatric symptom scores including OCD and tics scores, measured by psychological evaluations. This study demonstrates the use of immunological therapy to treat neuropsychiatric symptoms.

## Data Availability

The datasets presented in this article are not readily available because access to the data underlying this paper is tightly governed by various legislative and regulatory frameworks. De-identified clinical and laboratory data and response to treatment data for the study cohort included in this study can only be made available to legitimate researchers and clinicians from medical and academic institutions, for academic and clinical research upon request. A proposal with a detailed description of study objectives and a statistical analysis plan will be requested. The proposal will be evaluated based on data protection regulations and regulations about secondary use of patient data. After approval of a proposal, de-identified data will be shared through a secure online platform upon signing a data processing agreement. Requests to access the datasets should be directed to melamedi@immunoe.com.
