# Supplementary material for: IVIG response in pediatric acute-onset neuropsychiatric syndrome correlates with reduction in pro-inflammatory monocytes and neuropsychiatric measures
Source: Front Immunol. 2024 Oct 3;15:1383973. doi: 10.3389/fimmu.2024.1383973 (PMC11484259; doi:10.3389/fimmu.2024.1383973)

Supplementary materials

**Supplementary Table 1. Antibody panel**

All antibodies were purchased from BioLegend (San Diego, CA, USA) and were used at the concentration specified by the manufacturer. Together, the antibodies shown were used to identify monocytes, including a polarized subset, and dendritic cells in peripheral blood mononuclear cells.

| **Cell type** | **Marker and Flurophore** | **Function** |
| --- | --- | --- |
| Exclusion: T cell, B cell, granulocytes | PerCP Cy 5.5 anti-human CD3 (T cell) | CD3: antigen recognition and T cell activation.  CD19: antigen-independent development and Ig-induced activation of B cells.  CD66b: adhesion and activation of eosinophils  These cell-lineage markers were used to exclude these cells from the analysis |
|  | PerCP Cy5.5 anti-human CD19 (B cell) |  |
|  | PerCP Cy5.5 anti-human CD66b (granulocytes) |  |
| Monocyte markers | APC Cy7 anti-human HLA-DR | Antigen presentation to CD4 T cells |
|  | Pacific Blue anti-human CD14 | LPS receptor |
|  | Brilliant Violet BV650 anti-human CD16 | Receptor for the Fc portion of immunoglobulin G; also called FCyRIIIa |
|  | PECy5 anti-human CD11b | Pairs with CD18; mediates phagocytosis and cell adhesion and migration |
| Dendritic cell markers | Brilliant violet BV711 anti-human CD11c | Pairs with CD18; mediates cell adhesion (e.g., to fibrinogen), migration and phagocytosis |
|  | Brilliant Violet BV785 anti-human CD1c | Presentation of lipid-based antigens; on conventional myeloid dendritic cell subsets (DC2, DC3) |
| Pro-inflammatory, polarized monocyte | BUV805 anti-human CD64 | Receptor for Fc portion of immunoglobulin G, excluding the IgG2 subclass; also called FCyRI |
|  | FITC anti-human CD86 | Co-stimulatory molecule that binds to CD28 on T cells |

**Supplementary Table 2. Total white blood cell counts in patients with PANS following IVIG infusion**

| **Patient ID** | **WBC count** (× 10^3^/ml) | |
| --- | --- | --- |
|  | **Pre-IVIG**  **(Screening)** | **Post-IVIG**  **(Visit 8)** |
|  | 5900 | 5479 |
|  | 7800 | 6701 |
|  | 5500 | 5893 |
|  | 6900 | 7310 |
|  | 3600 | 4101 |
|  | 5500 | 5578 |
|  | 4500 | 4672 |
|  | 5400 | 6052 |
|  | 4100 | 3908 |
|  | 7100 | 6936 |

Each color represents a single patient and matches Figures 2 and 4.

IVIG, intravenous immunoglobulin; PANS, pediatric acute-onset neuropsychiatric syndrome; WBC, white blood cells

**Supplementary Figure 1: Gating strategy to identify pro-inflammatory CD14^+^ cells in fixed whole blood**

Representative contour plots showing the manual gating strategy to identify pro-inflammatory CD14^+^ cells in a patient with PANS at visit 1 of IVIG treatment. All samples were analyzed using the exact same gates. Axes labels are mentioned on the top right side of each plot and the gate names are indicated on the top right corner of the plot. Dump-gate was used to identify HLA-DR^+^ populations, which was further gated to identify CD14^+^ monocytes and pro-inflammatory CD14^+^ cells.


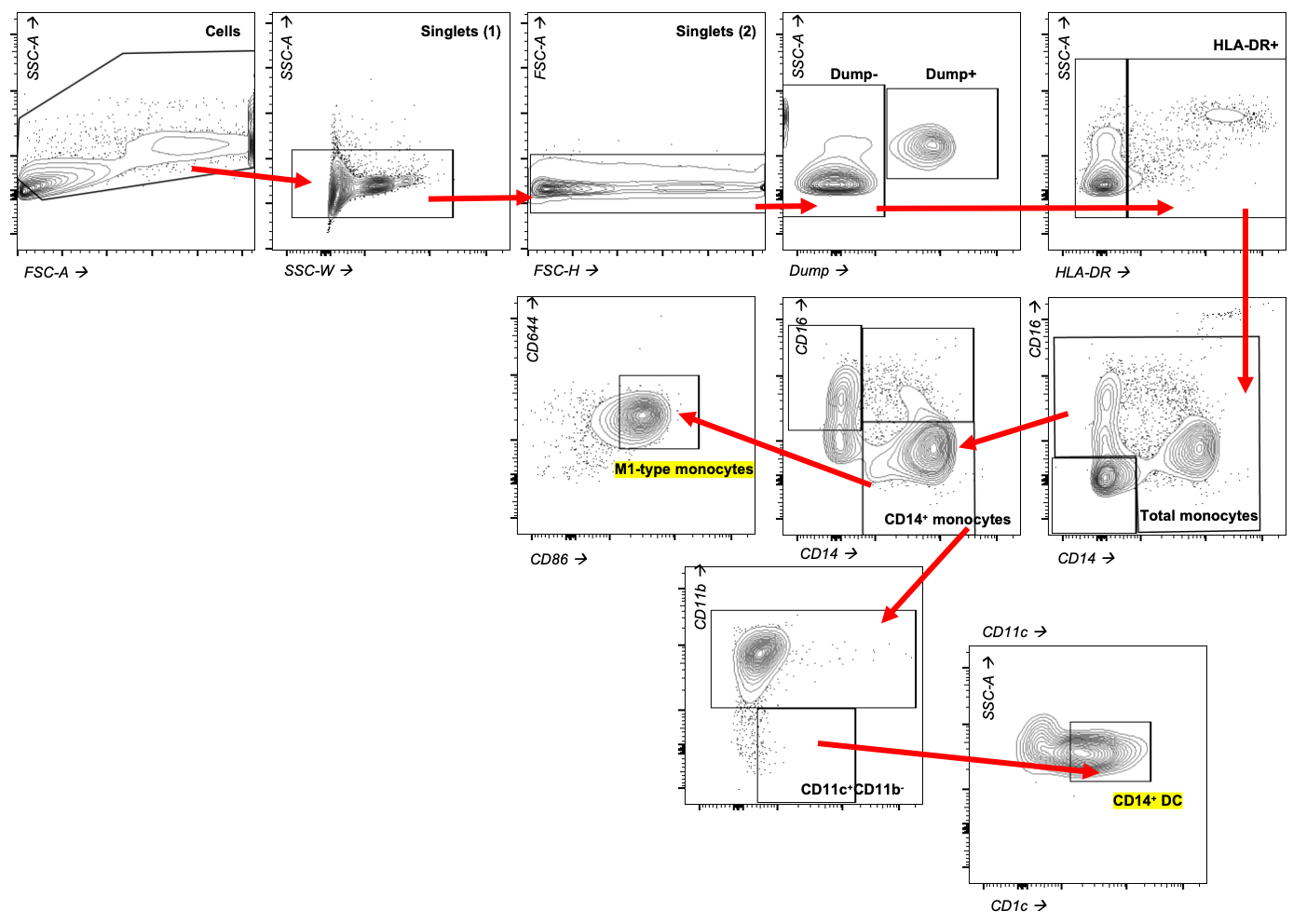

Supplement: Supplementary file 1 [file DataSheet1.docx]
